# Supplementary material for: Viromics Reveals the High Diversity of Viruses from Fishes of the Tibet Highland
Source: Microbiol Spectr. 2023 May 23;11(3):e00946-23. doi: 10.1128/spectrum.00946-23 (PMC10269613; doi:10.1128/spectrum.00946-23)

SUPPLEMENTAL MATERIAL

Viromics reveals the high diversity of viruses from fishes of the Tibet highland

Yuan Xi[1#], Xiaojie Jiang[1#], Xinrui Xie[2#], Min Zhao[1], Han Zhang[1], Kailin Qin[1], Xiaochun Wang[1], Yuwei Liu[1], Shixing Yang[1], Quan Shen[1], Likai Ji[1],

Peng Shang[2*], Wen Zhang[1*], Tongling Shan[3*]

1. Department of Microbiology, School of Medicine, Jiangsu University, Zhenjiang, 212013, Jiangsu, China
2. Animal Science College, Tibet Agriculture and Animal Husbandry University, Nyingchi, 860000, Tibet, China.
3. Shanghai Veterinary Research Institute, Chinese Academy of Agricultural Sciences, 200241, Shanghai, China.

* Corresponding author

E-mail: z0216wen@yahoo.com (WZ)

E-mail: nemoshpmh@126.com (PS)

E-mail: shantongling@shvri.ac.cn (TS)

# These authors contributed equally.

Table S1. Information of specific virus species in diffent pools

Table S2. Primers used for specific PCR confirmation and inverse PCR for novel papillomavirus

Table S3. Isolation of main viruses.

Fig S1. Phylogenetic relationship of Papillomaviridae. Phylogenetic tree based on E1 protein. The red name indicates the sequence obtained in this study. See legend for relevant labeling.

Table S1. Information of specific virus species in diffent pools

Pool	Virus species
Fish093	Macaca mulatta polyomavirus 1
	Po-Circo-like virus 41
	Bovismacovirus bovas2
	Chimpanzee associated porprismacovirus 1
	Howler monkey associated porprismacovirus 1
	Porcine associated porprismacovirus 5
	Porcine associated porprismacovirus 8
	Porprismacovirus bovas1
	PoSCV Kor J481
	Gila monster-associated gemycircularvirus
	CRESS virus sp.
	Human picobirnavirus
	Macaque picobirnavirus 25
	Infectious flacherie virus
	Posavirus 1
	Adelphocoris suturalis virus
	Beihai charybdis crab virus 1
	Hubei virga-like virus 21
	Hubei virga-like viurs 8
	Xingshan nematode virus 2
	Xinzhou nematode virus 1
	Brejeira virus
	Loreto virus

	Dragonfly-associated microphage 1
Fish094	Lepidopteran iteradensovirus 2<br>Densovirus SC3908<br>Parvovirus NIH-CQV<br>Circoviridae sp.<br>Porcine associated porprismacovirus 4<br>CRESS virus sp.<br>Picornavirales Q_sR_OV_020<br>Posavirus 1<br>Woolly monkey sarcoma virus<br>Dragonfly-associated microphage 1<br>McMurdo Ice Shelf pond-associated circular DNA virus-3
Fish095	Ambidensovirus sp.<br>Lepidopteran iteradensovirus 2<br>Blattodean pefuambidensovirus 1<br>Anseriform dependoparvovirus 1<br>Circoviridae sp.<br>Circovirus-like genome DHCV-6<br>Ecklonia radiata-associated virus 9<br>Porcine associated porprismacovirus 5<br>CRESS virus sp.<br>Severe acute respiratory syndrome-related coronavirus<br>Hubei chipolycivirus<br>Posavirus strain 8805<br>Sanxia water strider virus 16<br>Wuhan insect virus 8

Fish096	Torque teno rodent virus 2<br>Lepidopteran ambidensovirus 1<br>Ambidensovirus sp.<br>Lepidopteran iteradensovirus 2<br>Anseriform dependoparvovirus 1<br>Human associated circovirus 1<br>Bream circovirus<br>Circoviridae sp.<br>Circovirus-like genome DHCV-6<br>Hermit crab associated circular virus<br>CRESS virus sp.<br>Antarctic picorna-like virus 2<br>Avian leukosis virus<br>Avian sarcoma virus<br>Walleye epidermal hyperplasia virus 2<br>Murine leukemia virus<br>RD114 retrovirus<br>Citrus endogenous pararetrovirus<br>Beihai picorna-like virus 21<br>Dragonfly-associated microphage 1<br>synthetic Vaccinia virus
Fish097	Cyprinid herpesvirus 1<br>Cyprinid herpesvirus 2<br>Cyprinid herpesvirus 3<br>Chelonid alphaherpesvirus 5<br>Macropodid alphaherpesvirus 1

	Macacine betaherpesvirus 3<br>Equid gammaherpesvirus 5<br>Phocid gammaherpesvirus 3<br>Macacine gammaherpesvirus 8<br>Myotis gammaherpesvirus 8<br>Haliotid herpesvirus 1<br>Lepidopteran ambidensovirus 1<br>Ambidensovirus sp.<br>Lepidopteran iteradensovirus 3<br>Lepidopteran iteradensovirus 5<br>Lupine feces-associated densovirus 2<br>Ungulate bocaparvovirus 8<br>Anseriform dependoparvovirus 1<br>Carnivore protoparvovirus 1<br>Mink circovirus<br>Fur seal circovirus<br>Circoviridae sp.<br>Circovirus-like genome DHCV-6<br>Dromedary stool-associated circular ssDNA virus<br>CRESS virus sp.<br>Anticarsia gemmatalis multiple nucleopolyhedrovirus<br>Chrysodeixis chalcites nucleopolyhedrovirus<br>Pestivirus A<br>Pestivirus B<br>Norwalk virus<br>Infectious flacherie virus

	Megrivirus A
	Rous sarcoma virus
	Avian musculoaponeurotic fibrosarcoma virus AS42
	Avian sarcoma virus
	Walleye dermal sarcoma virus
	Walleye epidermal hyperplasia virus 1
	Walleye epidermal hyperplasia virus 2
	Feline leukemia virus
	Bat gammaretrovirus
	Murine leukemia virus
	Porcine type-C oncovirus
	Reticuloendotheliosis virus
	Galidia ERV
	Abelson murine leukemia virus
	Woolly monkey sarcoma virus
	Bovine immunodeficiency virus
	Human immunodeficiency virus 1
	Snakehead retrovirus
	Simian foamy virus
	Atlantic salmon swim bladder sarcoma virus
	Citrus endogenous pararetrovirus
	Human endogenous retrovirus W
	LNras*SN acutely transforming retrovirus
	Rhinella marina endogenous retrovirus
	Beihai noda-like virus 3
	Hubei picorna-like virus 60

	Sanxia picorna-like virus 1<br>Wenzhou picorna-like virus 10<br>Dragonfly larvae associated circular virus-2<br>Dragonfly-associated microphage 1<br>Nepavirus<br>Sewage-associated circular DNA virus-16<br>Canarypox virus<br>Turkeypox virus<br>Flamingopox virus FGPVKD09<br>Sea otterpox virus<br>Melanoplus sanguinipes entomopoxvirus<br>Duck atadenovirus A
Fish098	Anguillid herpesvirus 1<br>Human alphaherpesvirus 3<br>Monodontid alphaherpesvirus 1<br>Elephantid betaherpesvirus 1<br>Macaca mulatta polyomavirus 1<br>Ambidensovirus sp.<br>Lepidopteran iteradensovirus 1<br>Lepidopteran iteradensovirus 5<br>Densovirinae sp.<br>Densovirus SC116<br>Avon-Heathcote Estuary associated circular virus 3<br>Circoviridae sp.<br>Circovirus-like genome DHCV-6<br>Fur seal faeces associated circular DNA virus

	CRESS virus sp.<br>Anticarsia gemmatalis multiple nucleopolyhedrovirus<br>Hepacivirus C<br>Pestivirus A<br>Infectious flacherie virus<br>Avian leukosis virus<br>Avian musculoaponeurotic fibrosarcoma virus AS42<br>Avian sarcoma virus<br>Feline leukemia virus<br>Murine leukemia virus<br>Porcine type-C oncovirus<br>Galidia ERV<br>Equine foamy virus<br>Atlantic salmon swim bladder sarcoma virus<br>Citrus endogenous pararetrovirus<br>Rhinella marina endogenous retrovirus<br>Changjiang tombus-like virus 9<br>Sanxia picorna-like virus 1<br>Circovirus-like genome CB-A<br>Nepavirus<br>Diadromus pulchellus toursvirus<br>Myxoma virus<br>Salmon gillpox virus<br>BeAn 58058 virus<br>Melanoplus sanguinipes entomopoxvirus
Fish099	Anguillid herpesvirus 1

	Cyprinid herpesvirus 1<br>Cyprinid herpesvirus 3<br>Cercopithecine alphaherpesvirus 9<br>Equid gammaherpesvirus 5<br>Macacine gammaherpesvirus 5<br>Lepidopteran ambidensovirus 1<br>Lepidopteran iteradensovirus 3<br>Lepidopteran iteradensovirus 5<br>Danaus plexippus plexippus iteravirus<br>Dipteran protoambidensovirus 1<br>Hemipteran scindoambidensovirus 1<br>Orthopteran scindoambidensovirus 1<br>Densovirinae sp.<br>Densovirus SC116<br>Densovirus SC3908<br>Densovirus SC444<br>Densovirus SC525<br>Lone star tick densovirus 1<br>Lupine feces-associated densovirus 2<br>Viltain virus<br>Ungulate protoparvovirus 1<br>Parvovirus NIH-CQV<br>Bat associated circovirus 9<br>Beak and feather disease virus<br>Porcine circovirus 1<br>Rodent associated circovirus 6

	Bat circovirus<br>Circovirus sp.<br>Giant panda circovirus 1<br>Giant panda circovirus 4<br>Penaeus monodon circovirus VN11<br>Bat associated cyclovirus 10<br>Bat associated cyclovirus 15<br>Bovine associated cyclovirus 1<br>Dragonfly associated cyclovirus 4<br>Human associated cyclovirus 12<br>Human associated cyclovirus 4<br>Bat cyclovirus<br>Cyclovirus PKgoat21/PAK/2009<br>Cyclovirus ZM36a<br>Circoviridae 14 LDMD-2013<br>Circoviridae 16 LDMD-2013<br>Circoviridae 2 LDMD-2013<br>Circoviridae LDMD-2013b<br>Aiptasia sp. sea anemone associated circular virus<br>Avon-Heathcote Estuary associated circular virus 3<br>Avon-Heathcote Estuary associated circular virus 9<br>Circoviridae sp.<br>Circovirus-like genome DCCV-10<br>Circovirus-like genome DCCV-13<br>Circovirus-like genome DCCV-4<br>Circovirus-like genome DCCV-5

	Circovirus-like genome DCCV-7
	Circovirus-like genome DHCV-6
	Circovirus-like NI/2007-3
	Didemnum sp. Sea Squirt associated virus
	Dromedary stool-associated circular ssDNA virus
	Farfantepenaeus duorarum pink shrimp associated circular virus
	Gammarus sp. amphipod associated circular virus
	Hermit crab associated circular genome
	Hermit crab associated circular virus
	Human fecal virus Jorvi4
	Littorina sp. associated circular virus
	Marine snail associated circular virus
	Mytilus sp. clam associated circular virus
	Palaemonetes intermedius brackish grass shrimp associated circular virus
	Palaemonetes sp. common grass shrimp associated circular virus
	Pleurochrysis carterae circular virus
	Rodent circovirus
	CRESS virus sp.
	Agrotis ipsilon multiple nucleopolyhedrovirus
	Agrotis segetum nucleopolyhedrovirus B
	Chrysodeixis chalcites nucleopolyhedrovirus
	Lambdina fiscellaria nucleopolyhedrovirus
	Lymantria dispar multiple nucleopolyhedrovirus
	Perigonia lusca nucleopolyhedrovirus
	Spodoptera littoralis nucleopolyhedrovirus
	Spodoptera litura nucleopolyhedrovirus

	Trichoplusia ni single nucleopolyhedrovirus<br>Walleye dermal sarcoma virus<br>Walleye epidermal hyperplasia virus 1<br>Walleye epidermal hyperplasia virus 2<br>Feline leukemia virus<br>Porcine type-C oncovirus<br>Feline immunodeficiency virus<br>Atlantic salmon swim bladder sarcoma virus<br>Rhinella marina endogenous retrovirus<br>Ingleside virus<br>Circovirus-like genome CB-A<br>Ctenophore-associated circular virus 2<br>Cyanoramphus nest associated circular X DNA virus<br>Dragonfly larvae associated circular virus-2<br>Dragonfly larvae associated circular virus-3<br>Dragonfly-associated microphage 1<br>McMurdo Ice Shelf pond-associated circular DNA virus-3<br>McMurdo Ice Shelf pond-associated circular DNA virus-4<br>McMurdo Ice Shelf pond-associated circular DNA virus-6<br>Sewage-associated circular DNA virus-19<br>Sewage-associated circular DNA virus-20<br>Sewage-associated circular DNA virus-21<br>Sewage-associated circular DNA virus-29<br>Sewage-associated circular DNA virus-31<br>Sewage-associated circular DNA virus-32<br>Sewage-associated circular DNA virus-37

	Murmansk microtuspox virus<br>NY_014 poxvirus<br>Yokapox virus<br>Cowpox virus<br>Ectromelia virus<br>Skunkpox virus<br>Swinepox virus<br>Eptesipox virus
Fish100	Testudinid alphaherpesvirus 3<br>Macropodid alphaherpesvirus 1<br>Cercopithecine alphaherpesvirus 9<br>Equid gammaherpesvirus 5<br>Felid gammaherpesvirus 1<br>Phocid gammaherpesvirus 3<br>Ateline gammaherpesvirus 2<br>Macacine gammaherpesvirus 5<br>Macacine gammaherpesvirus 8<br>Myotis gammaherpesvirus 8<br>Papillomaviridae sp.<br>Lepidopteran ambidensovirus 1<br>Lepidopteran iteradensovirus 5<br>Dipteran protoambidensovirus 1<br>Densovirus SC116<br>Densovirus SC444<br>Linvill Road virus<br>Parvovirus NIH-CQV

	Bat associated circovirus 4
	Canine circovirus
	European catfish circovirus
	Asterias forbesi associated circular virus
	Bat circovirus
	Circovirus sp.
	Giant panda circovirus 1
	Giant panda circovirus 4
	Bat associated cyclovirus 15
	Dragonfly associated cyclovirus 2
	Human associated cyclovirus 4
	Circoviridae 14 LDMD-2013
	Circoviridae LDMD-2013b
	Aiptasia sp. sea anemone associated circular virus
	Avon-Heathcote Estuary associated circular virus 3
	Circoviridae sp.
	Circovirus-like genome DCCV-13
	Circovirus-like genome DHCV-6
	Circovirus-like NI/2007-3
	Dromedary stool-associated circular ssDNA virus
	Hermit crab associated circular virus
	Human fecal virus Jorvi4
	Marine snail associated circular virus
	Mytilus sp. clam associated circular virus
	Palaemonetes intermedius brackish grass shrimp associated circular virus
	Palaemonetes sp. common grass shrimp associated circular virus

	Pleurochrysis carterae circular virus
	Rodent circovirus
	CRESS virus sp.
	Lymantria dispar multiple nucleopolyhedrovirus
	Lymantria xylina nucleopolyhedrovirus
	Spodoptera littoralis nucleopolyhedrovirus
	Orthohepevirus D
	Bat hepevirus
	Wenling thamnaconus septentrionalis hepevirus
	Wenling thamnaconus striatus hepevirus
	Marmot picobirnavirus
	Guangdong pseudohemiculter dispar calicivirus
	Black queen cell virus
	Centovirus
	Antarctic picorna-like virus 2
	Nilaparvata lugens honeydew virus 1
	Helicoverpa armigera iflavirus
	Euscelidius variegatus virus 1
	Rhizosolenia setigera RNA virus 01
	Astarnavirus
	Jericarnavirus A
	Fur seal picorna-like virus
	Hubei chipolycivirus
	Picornavirales N_OV_001
	Robinvale bee virus 3
	Robinvale bee virus 8

	Bastrovirus BAS-3
	Heron hepatitis B virus
	Parrot hepatitis B virus
	White sucker hepatitis B virus
	Ingleside virus
	Beihai barnacle virus 4
	Beihai mollusks virus 1
	Beihai narna-like virus 10
	Beihai noda-like virus 3
	Beihai paphia shell virus 2
	Beihai picorna-like virus 107
	Beihai picorna-like virus 11
	Beihai picorna-like virus 14
	Beihai picorna-like virus 15
	Beihai picorna-like virus 20
	Beihai picorna-like virus 21
	Beihai picorna-like virus 28
	Beihai picorna-like virus 31
	Beihai picorna-like virus 43
	Beihai picorna-like virus 57
	Beihai picorna-like virus 7
	Beihai picorna-like virus 75
	Beihai picorna-like virus 8
	Beihai picorna-like virus 9
	Beihai sesarmid crab virus 1
	Beihai sipunculid worm virus 4

	Beihai tombus-like virus 6<br>Beihai tombus-like virus 7<br>Beihai tombus-like virus 8<br>Beihai zhaovirus-like virus 1<br>Beihai zhaovirus-like virus 4<br>Beihai zhaovirus-like virus 5<br>Changjiang crawfish virus 1<br>Changjiang narna-like virus 2<br>Changjiang tombus-like virus 13<br>Changjiang tombus-like virus 21<br>Hubei coleoptera virus 1<br>Hubei narna-like virus 8<br>Hubei odonate virus 3<br>Hubei partiti-like virus 24<br>Hubei picorna-like virus 1<br>Hubei picorna-like virus 26<br>Hubei picorna-like virus 31<br>Hubei picorna-like virus 61<br>Hubei tetragnatha maxillosa virus 2<br>Hubei tombus-like virus 12<br>Hubei tombus-like virus 36<br>Hubei tombus-like virus 9<br>Sanxia picorna-like virus 1<br>Sanxia picorna-like virus 2<br>Sanxia picorna-like virus 4<br>Sanxia picorna-like virus 5

	Sanxia picorna-like virus 8
	Sanxia water strider virus 16
	Sanxia water strider virus 7
	Shahe picorna-like virus 1
	Shahe picorna-like virus 2
	Shahe picorna-like virus 5
	Wenling picorna-like virus 1
	Wenling picorna-like virus 4
	Wenling tombus-like virus 1
	Wenzhou hepe-like virus 2
	Wenzhou noda-like virus 1
	Wenzhou picorna-like virus 1
	Wenzhou picorna-like virus 2
	Wenzhou picorna-like virus 4
	Wenzhou picorna-like virus 52
	Wenzhou picorna-like virus 6
	Wuhan millipede virus 3
	Chaetoceros tenuissimus RNA virus type-II
	Bat Middle East Hepe-Astrovirus
	Sclerophthora macrospora virus A
	Circovirus-like genome CB-A
	Dragonfly larvae associated circular virus-1
	Dragonfly larvae associated circular virus-2
	Dragonfly larvae associated circular virus-3
	Dragonfly-associated microphage 1
	McMurdo Ice Shelf pond-associated circular DNA virus-3

	McMurdo Ice Shelf pond-associated circular DNA virus-6<br>Nepavirus<br>Sewage-associated circular DNA virus-18<br>Sewage-associated circular DNA virus-19<br>Sewage-associated circular DNA virus-20<br>Sewage-associated circular DNA virus-21<br>Sewage-associated circular DNA virus-29<br>Sewage-associated circular DNA virus-32<br>Sewage-associated circular DNA virus-37<br>Heliothis virescens ascovirus 3i<br>Bovine mastadenovirus A<br>Dolphin mastadenovirus B
Fish101	Equid gammaherpesvirus 5<br>Lepidopteran ambidensovirus 1<br>Lepidopteran iteradensovirus 3<br>Lepidopteran iteradensovirus 5<br>Danaus plexippus plexippus iteravirus<br>Orthopteran scindoambidensovirus 1<br>Porcine parvovirus 2<br>Ungulate protoparvovirus 1<br>Parvovirus NIH-CQV<br>Bat circovirus<br>Circovirus sp.<br>Giant panda circovirus 1<br>Bat associated cyclovirus 15<br>Human associated cyclovirus 4

	Circoviridae 16 LDMD-2013
	Circoviridae LDMD-2013b
	Circoviridae sp.
	Circovirus-like genome DHCV-6
	Dromedary stool-associated circular ssDNA virus
	Ecklonia radiata-associated virus 2
	Hermit crab associated circular genome
	Hermit crab associated circular virus
	Palaemonetes intermedius brackish grass shrimp associated circular virus
	Pleurochrysis carterae circular virus
	Gemygorvirus ptero1
	CRESS virus sp.
	Aphis glycines virus 3
	Centovirus
	Antarctic picorna-like virus 2
	Armigeres iflavirus
	Nasonia vitripennis virus
	Culex Iflavi-like virus 2
	Euscelidius variegatus virus 1
	Yongsan iflavirus 1
	Chaetoceros tenuissimus RNA virus 01
	Rhizosolenia setigera RNA virus 01
	Astarnavirus
	Chaetarnavirus 2
	Jericarnavirus A
	Fur seal picorna-like virus

	Hubei chipolycivirus
	Barns Ness breadcrumb sponge aquatic picorna-like virus 1
	Picornavirales N_OV_001
	Robinvale bee virus 3
	Robinvale bee virus 9
	White sucker hepatitis B virus
	Ingleside virus
	Leptomonas pyrrhocoris RNA virus
	Beihai mollusks virus 1
	Beihai noda-like virus 3
	Beihai octopus virus 1
	Beihai paphia shell virus 2
	Beihai picorna-like virus 107
	Beihai picorna-like virus 11
	Beihai picorna-like virus 14
	Beihai picorna-like virus 15
	Beihai picorna-like virus 17
	Beihai picorna-like virus 20
	Beihai picorna-like virus 21
	Beihai picorna-like virus 28
	Beihai picorna-like virus 31
	Beihai picorna-like virus 4
	Beihai picorna-like virus 57
	Beihai picorna-like virus 6
	Beihai picorna-like virus 7
	Beihai picorna-like virus 8

	Beihai picorna-like virus 9<br>Beihai sesarmid crab virus 1<br>Beihai sipunculid worm virus 4<br>Beihai tombus-like virus 13<br>Beihai tombus-like virus 8<br>Beihai zhaovirus-like virus 1<br>Beihai zhaovirus-like virus 4<br>Beihai zhaovirus-like virus 5<br>Changjiang tombus-like virus 1<br>Changjiang tombus-like virus 13<br>Changjiang tombus-like virus 21<br>Changjiang tombus-like virus 9<br>Hubei narna-like virus 8<br>Hubei picorna-like virus 1<br>Hubei picorna-like virus 18<br>Hubei picorna-like virus 61<br>Hubei sobemo-like virus 2<br>Hubei tombus-like virus 12<br>Hubei tombus-like virus 25<br>Hubei tombus-like virus 31<br>Hubei tombus-like virus 36<br>Hubei tombus-like virus 9<br>Hubei virga-like virus 11<br>Hubei virga-like virus 12<br>Sanxia picorna-like virus 1<br>Sanxia picorna-like virus 2

	Sanxia picorna-like virus 4<br>Sanxia picorna-like virus 5<br>Sanxia picorna-like virus 8<br>Sanxia water strider virus 16<br>Shahe picorna-like virus 1<br>Shahe picorna-like virus 11<br>Shahe picorna-like virus 2<br>Shahe picorna-like virus 5<br>Wenling picorna-like virus 1<br>Wenzhou noda-like virus 1<br>Wenzhou picorna-like virus 1<br>Wenzhou picorna-like virus 2<br>Wenzhou picorna-like virus 52<br>Wenzhou picorna-like virus 6<br>Wenzhou picorna-like virus 8<br>Wuhan millipede virus 3<br>Xinzhou nematode virus 1<br>Boutonnet virus<br>Chaetoceros tenuissimus RNA virus type-II<br>Ngewotan negevirus<br>Sclerophthora macrospora virus A<br>Dragonfly larvae associated circular virus-2<br>Dragonfly larvae associated circular virus-3<br>Dragonfly-associated microphage 1<br>McMurdo Ice Shelf pond-associated circular DNA virus-3<br>McMurdo Ice Shelf pond-associated circular DNA virus-6

	Nepavirus<br>Sewage-associated circular DNA virus-19<br>Sewage-associated circular DNA virus-21<br>Sewage-associated circular DNA virus-32
Fish102	Equid gammaherpesvirus 5<br>Lepidopteran ambidensovirus 1<br>Lepidopteran iteradensovirus 5<br>Porcine parvovirus 2<br>Parvovirus NIH-CQV<br>Bat circovirus<br>Giant panda circovirus 1<br>Human associated cyclovirus 4<br>Circoviridae LDMD-2013b<br>Circoviridae sp.<br>Circovirus-like genome DHCV-6<br>Ecklonia radiata-associated virus 2<br>Hermit crab associated circular virus<br>Pleurochrysis carterae circular virus<br>CRESS virus sp.<br>Centovirus<br>Antarctic picorna-like virus 2<br>Deformed wing virus<br>Dinocampus coccinellae paralysis virus<br>Nilaparvata lugens honeydew virus 1<br>Armigeres iflavirus<br>Helicoverpa armigera iflavirus

	Chequa iflavirus
	Euscelidius variegatus virus 1
	Chaetoceros tenuissimus RNA virus 01
	Rhizosolenia setigera RNA virus 01
	Jericarnavirus A
	Fur seal picorna-like virus
	Washington bat picornavirus
	Hubei chipolycivirus
	Picornavirales N_OV_001
	Picornavirales N_OV_013
	Robinvale bee virus 3
	Robinvale bee virus 9
	Beihai barnacle virus 4
	Beihai mollusks virus 1
	Beihai noda-like virus 3
	Beihai octopus virus 1
	Beihai paphia shell virus 2
	Beihai picorna-like virus 107
	Beihai picorna-like virus 11
	Beihai picorna-like virus 14
	Beihai picorna-like virus 15
	Beihai picorna-like virus 20
	Beihai picorna-like virus 21
	Beihai picorna-like virus 28
	Beihai picorna-like virus 31
	Beihai picorna-like virus 4

	Beihai picorna-like virus 57
	Beihai picorna-like virus 6
	Beihai picorna-like virus 7
	Beihai picorna-like virus 8
	Beihai picorna-like virus 9
	Beihai sesarmid crab virus 1
	Beihai sipunculid worm virus 4
	Beihai tombus-like virus 8
	Beihai zhaovirus-like virus 1
	Beihai zhaovirus-like virus 4
	Beihai zhaovirus-like virus 5
	Changjiang tombus-like virus 13
	Changjiang tombus-like virus 21
	Changjiang tombus-like virus 9
	Hubei coleoptera virus 1
	Hubei narna-like virus 8
	Hubei odonate virus 3
	Hubei picorna-like virus 1
	Hubei picorna-like virus 26
	Hubei picorna-like virus 31
	Hubei picorna-like virus 61
	Hubei tetragnatha maxillosa virus 2
	Hubei tombus-like virus 12
	Hubei tombus-like virus 31
	Hubei tombus-like virus 36
	Sanxia noda-like virus 1

	Sanxia picorna-like virus 1<br>Sanxia picorna-like virus 2<br>Sanxia picorna-like virus 4<br>Sanxia picorna-like virus 5<br>Sanxia water strider virus 16<br>Shahe heteroptera virus 1<br>Shahe picorna-like virus 1<br>Shahe picorna-like virus 2<br>Shahe picorna-like virus 5<br>Wenling picorna-like virus 1<br>Wenzhou noda-like virus 1<br>Wenzhou picorna-like virus 1<br>Wenzhou picorna-like virus 2<br>Wenzhou picorna-like virus 52<br>Wenzhou picorna-like virus 6<br>Wuhan millipede virus 3<br>Chaetoceros tenuissimus RNA virus type-II<br>Sclerophthora macrospora virus A<br>Dragonfly larvae associated circular virus-2<br>Dragonfly-associated microphage 1<br>McMurdo Ice Shelf pond-associated circular DNA virus-3<br>McMurdo Ice Shelf pond-associated circular DNA virus-6<br>Sewage-associated circular DNA virus-19<br>Sewage-associated circular DNA virus-21<br>Sewage-associated circular DNA virus-32
Fish103	Hemipteran ambidensovirus 2

	Lepidopteran ambidensovirus 1<br>Ambidensovirus sp.<br>Hemipteran diciambidensovirus 1<br>Lepidopteran iteradensovirus 1<br>Lepidopteran iteradensovirus 2<br>Lepidopteran iteradensovirus 3<br>Lepidopteran iteradensovirus 5<br>Danaus plexippus plexippus iteravirus<br>Densovirinae sp.<br>Densovirus SC3908<br>Human CSF-associated densovirus<br>Lone star tick densovirus 1<br>Lupine feces-associated densovirus 2<br>Porcine parvovirus 2<br>Ungulate protoparvovirus 1<br>Parvovirus NIH-CQV<br>Starling circovirus<br>Bat circovirus<br>Circoviridae sp.<br>Palaemonetes intermedius brackish grass shrimp associated circular virus<br>CRESS virus sp.<br>Pariacoto virus<br>Lunovirus<br>Ancient Northwest Territories cripavirus<br>Dicistroviridae sp.<br>Deformed wing virus

	Nilaparvata lugens honeydew virus 1<br>Armigeres iflavirus<br>Helicoverpa armigera iflavirus<br>Culex Iflavi-like virus 2<br>Yongsan iflavirus 1<br>Hubei chipolycivirus<br>Beihai barnacle virus 4<br>Beihai levi-like virus 33<br>Beihai picorna-like virus 107<br>Hubei coleoptera virus 1<br>Hubei noda-like virus 7<br>Hubei noda-like virus 9<br>Hubei odonate virus 3<br>Hubei picorna-like virus 26<br>Hubei tetragnatha maxillosa virus 2<br>Hubei tombus-like virus 31<br>Sanxia water strider virus 16<br>Sanxia water strider virus 17<br>Sanxia water strider virus 7<br>Shahe hepe-like virus 1<br>Shahe hepe-like virus 2<br>Shahe narna-like virus 2<br>Shahe picorna-like virus 11<br>Wenling levi-like virus 2<br>Dragonfly larvae associated circular virus-3<br>Dragonfly-associated microphage 1

	Porcine serum-associated circular virus<br>Sewage-associated circular DNA virus-21<br>Sewage-associated circular DNA virus-26<br>Sewage-associated circular DNA virus-29<br>Sewage-associated circular DNA virus-30
Fish104	Lepidopteran ambidensovirus 1<br>Ambidensovirus sp.<br>Lepidopteran iteradensovirus 3<br>Lepidopteran iteradensovirus 5<br>Danaus plexippus plexippus iteravirus<br>Densovirinae sp.<br>Human CSF-associated densovirus<br>Lone star tick densovirus 1<br>Lupine feces-associated densovirus 2<br>Galliform aveparvovirus 1<br>Porcine parvovirus 2<br>Ungulate protoparvovirus 1<br>Parvovirus NIH-CQV<br>Bat circovirus<br>Circoviridae sp.<br>CRESS virus sp.<br>Nilaparvata lugens honeydew virus 1<br>Helicoverpa armigera iflavirus<br>Hubei chipolycivirus<br>Robinvale bee virus 8<br>Beihai picorna-like virus 100

	Beihai picorna-like virus 101<br>Beihai picorna-like virus 107<br>Changjiang crawfish virus 5<br>Changjiang sobemo-like virus 2<br>Hubei coleoptera virus 1<br>Hubei odonate virus 3<br>Hubei picorna-like virus 60<br>Hubei tetragnatha maxillosa virus 2<br>Hubei tombus-like virus 25<br>Shahe hepe-like virus 1<br>Ubei picorna-like virus 3<br>Wenzhou narna-like virus 9<br>Wenzhou picorna-like virus 34<br>Wenzhou picorna-like virus 35<br>Wuhan fly virus 6<br>Dragonfly-associated microphage 1<br>McMurdo Ice Shelf pond-associated circular DNA virus-6<br>Sewage-associated circular DNA virus-21
Fish105	Anguillid herpesvirus 1<br>Cyprinid herpesvirus 1<br>Cyprinid herpesvirus 3<br>Ictalurid herpesvirus 2<br>Chelonid alphaherpesvirus 5<br>Testudinid alphaherpesvirus 3<br>Macropodid alphaherpesvirus 1<br>Bovine alphaherpesvirus 5

	Human alphaherpesvirus 3
	Monodontid alphaherpesvirus 1
	Cercopithecine betaherpesvirus 5
	Elephant endotheliotropic herpesvirus 4
	Bovine gammaherpesvirus 6
	Equid gammaherpesvirus 2
	Equid gammaherpesvirus 5
	Felid gammaherpesvirus 1
	Bovine gammaherpesvirus 4
	Macacine gammaherpesvirus 5
	Macaca mulatta polyomavirus 1
	Densovirus SC525
	Camel bocavirus 3
	Carnivore protoparvovirus 1
	Chiropteran protoparvovirus 1
	Eulipotyphla protoparvovirus 1
	Primate protoparvovirus 3
	Rodent protoparvovirus 1
	Rodent protoparvovirus 3
	Bat parvovirus SC630
	Rat minute virus 2a
	Ungulate protoparvovirus 1
	Parvovirus NIH-CQV
	Sea otter parvovirus 1
	Bat associated circovirus 3
	Beak and feather disease virus

	European catfish circovirus<br>Gull circovirus<br>Human associated circovirus 1<br>Anguilla anguilla circovirus<br>Asterias forbesi associated circular virus<br>Bat circovirus<br>Giant panda circovirus 1<br>Bat associated cyclovirus 10<br>Dragonfly associated cyclovirus 4<br>Horse associated cyclovirus 1<br>Human associated cyclovirus 4<br>Human associated cyclovirus 8<br>Bat cyclovirus<br>Cyclovirus PKgoat21/PAK/2009<br>Cyclovirus sp.<br>Cyclovirus ZM36a<br>Aiptasia sp. sea anemone associated circular virus<br>Circoviridae sp.<br>Circovirus-like genome DHCV-1<br>Circovirus-like genome DHCV-6<br>Ecklonia radiata-associated virus 2<br>Hermit crab associated circular virus<br>Marine snail associated circular virus<br>Gemycircularvirus furse1<br>CRESS virus sp.<br>Leucania separata nucleopolyhedrovirus

	Lymantria dispar multiple nucleopolyhedrovirus<br>Spodoptera litura nucleopolyhedrovirus<br>Peridroma alphabaculovirus<br>Cryptophlebia leucotreta granulovirus<br>Cydia pomonella granulovirus<br>Phthorimaea operculella granulovirus<br>Plutella xylostella granulovirus<br>Neodiprion lecontei nucleopolyhedrovirus<br>Neodiprion sertifer nucleopolyhedrovirus<br>Wenling thamnaconus striatus hepevirus<br>Chinese softshell turtle hepacivirus<br>Wenling shark virus<br>Pestivirus A<br>Pestivirus B<br>Nordland virus<br>Guangdong pseudohemiculter dispar calicivirus<br>Dicistroviridae sp.<br>Antarctic picorna-like virus 2<br>Infectious flacherie virus<br>Avian leukosis virus<br>Rous sarcoma virus<br>Avian carcinoma virus<br>Avian musculoaponeurotic fibrosarcoma virus AS42<br>Avian retrovirus<br>Avian sarcoma virus<br>Lymphoproliferative disease virus

	Y73 sarcoma virus
	Walleye dermal sarcoma virus
	Walleye epidermal hyperplasia virus 1
	Walleye epidermal hyperplasia virus 2
	Feline leukemia virus
	Gibbon ape leukemia virus
	Hardy-Zuckerman feline sarcoma virus
	Koala retrovirus
	Bat gammaretrovirus
	Murine leukemia virus
	Porcine type-C oncovirus
	Reticuloendotheliosis virus
	Echidna ERV
	Galidia ERV
	Abelson murine leukemia virus
	Baboon endogenous virus
	Murine leukemia-related retroviruses
	Murine osteosarcoma virus
	RD114 retrovirus
	Woolly monkey sarcoma virus
	Equine infectious anemia virus
	Feline immunodeficiency virus
	Human immunodeficiency virus 1
	Simian immunodeficiency virus
	Snakehead retrovirus
	Bovine foamy virus

	Equine foamy virus
	Feline foamy virus
	Puma feline foamy virus
	Bornean orangutan simian foamy virus
	Japanese macaque simian foamy virus
	Spider monkey simian foamy virus
	Squirrel monkey simian foamy virus
	Simian foamy virus
	Molossus molossus foamy virus 1
	AKT8 retrovirus
	Atlantic salmon swim bladder sarcoma virus
	Citrus endogenous pararetrovirus
	Human endogenous retrovirus W
	IC4 retrovirus
	LNras*SN acutely transforming retrovirus
	Rhinella marina endogenous retrovirus
	Rhinolophus affinis foamy virus 1
	Python curtus endogenous retrovirus
	Beihai picorna-like virus 14
	Hubei coleoptera virus 1
	Sanxia water strider virus 16
	Dragonfly-associated microphage 1
	McMurdo Ice Shelf pond-associated circular DNA virus-6
	Spodoptera frugiperda ascovirus 1a
	Canarypox virus
	Penguinpox virus

	Turkeypox virus<br>Flamingopox virus FGPVKD09<br>Goatpox virus<br>Sheeppox virus<br>Mule deerpox virus<br>Cowpox virus<br>Skunkpox virus<br>Vaccinia virus<br>Swinepox virus<br>White-tailed deer poxvirus<br>Eptesipox virus<br>Yaba monkey tumor virus<br>Melanoplus sanguinipes entomopoxvirus<br>Duck atadenovirus A
Fish106	Ungulate protoparvovirus 1<br>Giant panda circovirus 1<br>Circoviridae sp.<br>Circovirus-like genome DHCV-6<br>Porcine associated porprismacovirus 4<br>Porcine associated porprismacovirus 5<br>Porcine associated porprismacovirus 8<br>Wenling thamnaconus striatus hepevirus<br>Norwalk virus<br>Walleye dermal sarcoma virus<br>Walleye epidermal hyperplasia virus 1<br>Walleye epidermal hyperplasia virus 2

	Feline leukemia virus<br>Woolly monkey sarcoma virus<br>Human immunodeficiency virus 1<br>Bovine foamy virus<br>Rhinella marina endogenous retrovirus<br>Hubei odonate virus 3<br>Dragonfly-associated microphage 1
Fish107	Cyprinid herpesvirus 2<br>Cyprinid herpesvirus 3<br>Chelonid alphaherpesvirus 5<br>Human alphaherpesvirus 1<br>Lepidopteran iteradensovirus 5<br>Anseriform dependoparvovirus 1<br>Circoviridae sp.<br>Circovirus-like genome DHCV-6<br>Hermit crab associated circular virus<br>Rat associated porprismacovirus 1<br>Infectious flacherie virus<br>Nilaparvata lugens honeydew virus 1<br>Spodoptera exigua iflavirus 1<br>Hubei chipolycivirus<br>Avian sarcoma virus<br>Y73 sarcoma virus<br>Walleye dermal sarcoma virus<br>Walleye epidermal hyperplasia virus 1<br>Walleye epidermal hyperplasia virus 2

Fish108	Equid gammaherpesvirus 2<br>Circoviridae sp.<br>Circovirus-like genome DHCV-6<br>Po-Circo-like virus<br>Porcine associated porprismacovirus 9<br>CRESS virus sp.<br>Lambdina fiscellaria nucleopolyhedrovirus<br>Mocis latipes granulovirus<br>Infectious flacherie virus<br>Avian leukosis virus<br>Avian carcinoma virus<br>Avian retrovirus IC10<br>Avian sarcoma virus<br>Feline leukemia virus<br>Atlantic salmon swim bladder sarcoma virus<br>Citrus endogenous pararetrovirus<br>Rhinella marina endogenous retrovirus<br>Anomala cuprea entomopoxvirus<br>Duck atadenovirus A
Fish109	Gallid alphaherpesvirus 1<br>Gallid alphaherpesvirus 2<br>Chelonid alphaherpesvirus 5<br>Monodontid alphaherpesvirus 1<br>Elephant endotheliotropic herpesvirus 4<br>Bovine gammaherpesvirus 4<br>Human gammaherpesvirus 8

	Macacine gammaherpesvirus 5
	Macaca mulatta polyomavirus 1
	Ambidensovirus sp.
	Anseriform dependoparvovirus 1
	Circoviridae sp.
	Circovirus-like genome DHCV-6
	CRESS virus sp.
	Lambdina fiscellaria nucleopolyhedrovirus
	Neodiprion sertifer nucleopolyhedrovirus
	Hepacivirus C
	Infectious flacherie virus
	Posavirus 1
	Avian leukosis virus
	Avian musculoaponeurotic fibrosarcoma virus AS42
	Avian retrovirus IC10
	Avian sarcoma virus
	Y73 sarcoma virus
	Walleye dermal sarcoma virus
	Walleye epidermal hyperplasia virus 2
	Feline leukemia virus
	Koala retrovirus
	Bat gammaretrovirus
	Murine leukemia virus
	Echidna ERV
	Bovine immunodeficiency virus
	Human immunodeficiency virus 1

	Snakehead retrovirus<br>Atlantic salmon swim bladder sarcoma virus<br>Citrus endogenous pararetrovirus<br>Human endogenous retrovirus W<br>Rhinella marina endogenous retrovirus<br>Hubei virga-like virus 23<br>Diadromus pulchellus toursvirus<br>African swine fever virus<br>Canarypox virus<br>Yokapox virus<br>Camelpox virus<br>Pseudocowpox virus<br>Squirrelpox virus<br>Eptesipox virus<br>Tanapox virus<br>Choristoneura biennis entomopoxvirus<br>Melanoplus sanguinipes entomopoxvirus
Fish110	Chelonid alphaherpesvirus 5<br>Ambidensovirus sp.<br>Anseriform dependoparvovirus 1<br>Porcine associated porprismacovirus 5<br>CRESS virus sp.<br>Mamestra configurata nucleopolyhedrovirus A<br>Hepacivirus C<br>Infectious flacherie virus<br>Megrivirus A

	Posavirus 1
	Avian carcinoma virus
	Avian sarcoma virus
	Walleye dermal sarcoma virus
	Walleye epidermal hyperplasia virus 1
	Walleye epidermal hyperplasia virus 2
	Feline leukemia virus
	Murine leukemia virus
	Porcine type-C oncovirus
	Reticuloendotheliosis virus
	Galidia ERV
	Human immunodeficiency virus 1
	Equine foamy virus
	Spider monkey simian foamy virus
	Simian foamy virus
	Molossus molossus foamy virus 1
	Atlantic salmon swim bladder sarcoma virus
	Citrus endogenous pararetrovirus
	Rhinella marina endogenous retrovirus
	Rhinolophus affinis foamy virus 1
	Dragonfly-associated microphage 1
	Eptesipox virus
	Anomala cuprea entomopoxvirus
	Melanoplus sanguinipes entomopoxvirus

Table S2. Primers used for specific PCR confirmation and inverse PCR for novel papillomavirus

Primer	Targeted virus	Application	Sequence (5´-3´)	Fragment size (bp)	Annealing temperature (℃)	No. of cycles
Pvw sense	papillomavirus	PCR confirmation	TGAAGAAACAGAGCCAGAGTCT	743	50	35
Pvw antisense			ACCAGGCTGCTATATCATCCAC			
Pvn sense			GGAAAGCAACAGTTCGTGGC	606	55	35
Pvn antisense			ACCAGGCTGCTATATCATCCAC			
Hevw sense	hepevirus	PCR confirmation	AGATGACCCTTTGACCGCTG	754	55	35
Hevw antisense			TGCATCCGATGATCTCCACG			
Hevn sense			TGTGCTCTTCTGCTTCGGAG	582	58	35
Hevn antisense			CCACGGCACATTTCGACAAG			
Havw sense	hepadnavirus	PCR confirmation	TCACATCCCAATGGACGTCG	842	55	35
Havw antisense			CGCTTGTAAGAGCATGTCGC			
Havn sense			GGACGATTTCCTCCTCGCAC	451	58	35
Havn antisense			TGTCGATCCTGAATCCGCTG			
PVWF	papillomavirus	Inverse PCR	TCTGAGCCTGATCTTTCTGCA	1001	50	35
PVWR			CCAATGTATGCAGGCAAGGT			
PVNF			TGTCTGTATGGCAGGTGACAG	935	55	35
PVNR			GCCACGAACTGTTGCTTTCC			

Table S3. Isolation of main viruses.

Targeted virus	Pool	Family	Body parts
papillomavirus	Fish094	Cyprinidae	Gill
	Fish100	Cyprinidae	Intestinal contents
	Fish106	Cyprinidae	liver, muscle, swim bladder, heart, brain
hepevirus	Fish100	Cyprinidae	Intestinal contents
	Fish106	Cyprinidae	liver, muscle, heart, brain
hepadnavirus	Fish093	Cyprinidae	Gill
	Fish094	Cyprinidae	Gill
	Fish095	Cyprinidae	Gill
	Fish096	Cyprinidae	Gill
	Fish099	Cyprinidae	Intestinal contents
	Fish100	Cyprinidae	Intestinal contents
	Fish101	Cyprinidae	Intestinal contents
	Fish102	Cyprinidae	Intestinal contents
	Fish105	Cyprinidae	liver, muscle, swim bladder, brain
	Fish106	Cyprinidae	liver, muscle, heart, brain
	Fish107	Cyprinidae	liver, muscle, swim bladder, brain

Fig S1. Phylogenetic relationship of Papillomaviridae. Phylogenetic tree based on E1 protein. The red name indicates the sequence obtained in this study. See legend for relevant labeling.

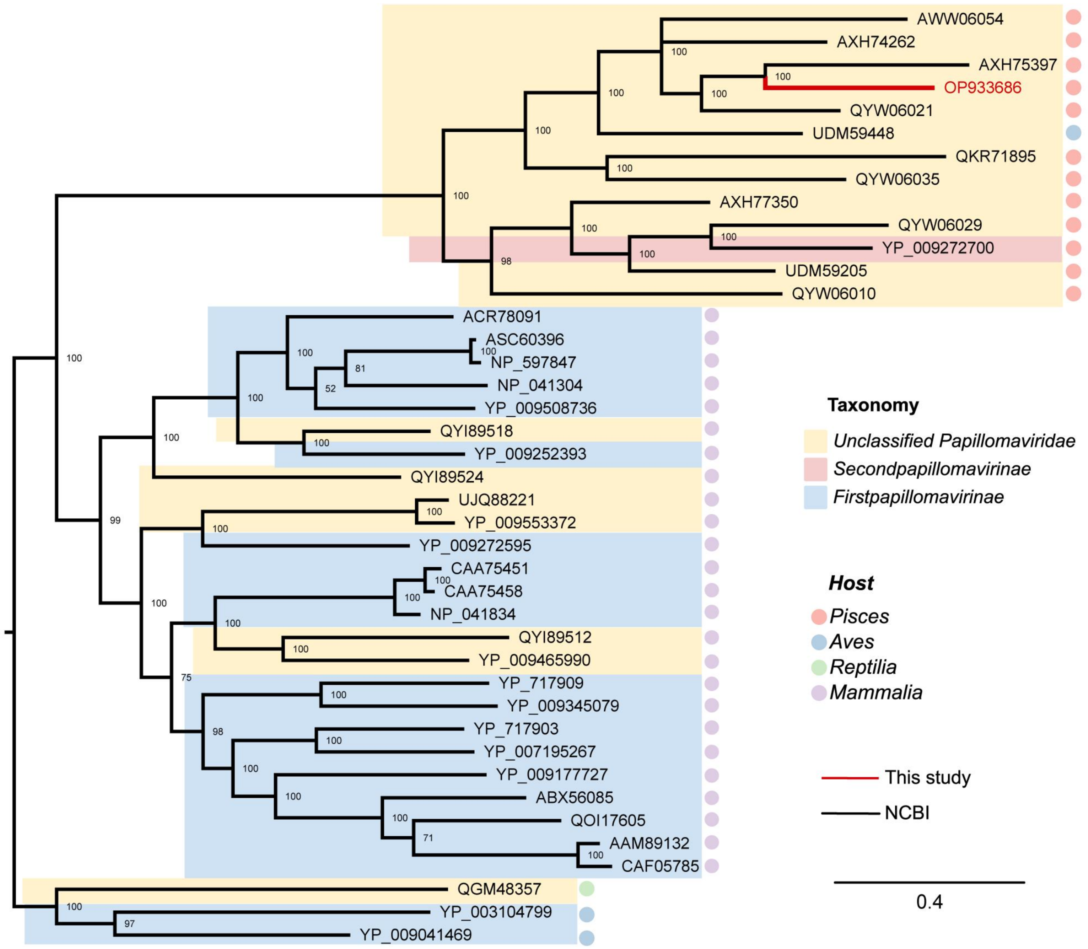

Supplement: Supplemental file 1 — Supplemental material. Download spectrum.00946-23-s0001.pdf, PDF file, 0.5 MB [file spectrum.00946-23-s0001.pdf]
